# Synthesis, Structure, and Tunability of Zero-Dimensional Organic–Inorganic Metal Halides Utilizing the *m*-Xylylenediammonium Cation: MXD_2_PbI_6_, MXDBiI_5_, and MXD_3_Bi_2_Br_12_·2H_2_O

**DOI:** 10.1021/acs.cgd.2c00187

**Published:** 2022-04-28

**Authors:** Pia S. Klee, Yuri Hirano, David B. Cordes, Alexandra M. Z. Slawin, Julia L. Payne

**Affiliations:** † School of Chemistry, University of St Andrews, North Haugh, St Andrews, Fife KY16 9ST, United Kingdom

## Abstract

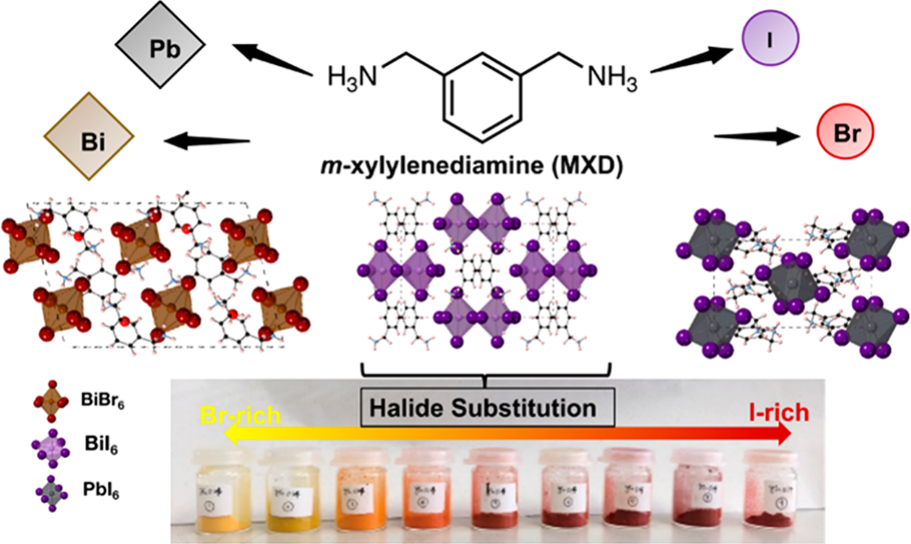

Over the past decade, the efficiency of photovoltaic devices based on CH_3_NH_3_PbI_3_ have dramatically increased. This has driven research efforts in all areas, from the discovery of materials to film processing to long-term device stability studies. Here, we report the synthesis and structure of three new “zero dimensional” organic–inorganic metal halides which use the meta-xylylenediammonium (MXD) cation: MXD_2_PbI_6_, MXDBiI_5_, and (MXD)_3_Bi_2_Br_12_·2H_2_O. The different structures of the new materials lead to compounds with a range of band gaps with MXDBiI_5_ having the lowest at 2.15 eV. We have explored the tunabilty of MXDBiI_5_ through halide substitution by preparing a series of samples with composition MXDBiI_5–*x*_Br_*x*_ and determined the halide content using energy dispersive X-ray spectroscopy. A large range of solid solution is obtained in MXDBiI_5–*x*_Br_*x*_, resulting in the formation of single-phase materials for bromine contents from *x* = 0 to 3.71 (iodine contents from 1.29 to 5). This highlights the fact that zero-dimensional organic–inorganic halides are highly tunable, in a similar manner to the higher-dimensional perovskite counterparts. Such new materials open up the opportunity for further studies of the physics and optoelectronic properties of these materials.

## Abstract 2

The structures of MXD_2_PbI_6_, MXDBiI_5_ and MXD_3_Bi_2_Br_12_·2H_2_O have been determined. Halide substitution has been explored in MXDBiI_5−*x*_Br_*x*_, and a large region of solid solution has been found. MXDBiI_5_ has short intercluster I−I distances and π−π stacking between the MXD cations while also possessing the lowest band gap of the materials studied here.

## Introduction

In 2009, Miyasaka and co-workers first tested CH_3_NH_3_PbI_3_ and CH_3_NH_3_PbBr_3_ in photovoltaic devices.^1^ Although the initial efficiencies were modest, the resulting optimization of all aspects of device fabrication has led to groundbreaking photovoltaic power conversion efficiencies.^2−4^ Power conversion efficiencies of 25.7% have now been reported for single junction photovoltaics based on CH_3_NH_3_PbI_3_ and 29.8% for tandem photovoltaics, based on a combination of silicon and a perovskite layer.^5^

CH_3_NH_3_PbI_3_ adopts the perovskite structure, (ABX_3_) where A = CH_3_NH_3_^+^, B= Pb^2+^, and X = I^–^ (or another halide). The structure consists of corner-sharing PbI_6_ octahedra, with the organic cation being disordered on the perovskite A-site at room temperature.^6^ As the size of the organic ammonium cation is increased, different perovskite-related structure types can form.^7^ In particular, layered perovskites based on the Ruddlesden–Popper and Dion–Jacobson structure types have received considerable interest. A notable breakthrough came when a power conversion efficiency of 12.52% was achieved for (C_4_H_9_NH_3_)_2_(CH_3_NH_3_)_3_Pb_4_I_13_, which adopts the Ruddlesden–Popper structure.^8^ Very recently, the “Memory Seed Effect” has been reported, which involves dissolving presynthesized crystals in solvents such as DMF, which enables the preparation of high-quality thin films for a variety of layered perovskites.^9^ The solutions have been shown to retain “memory seed” crystallites, which facilitates the production of phase-pure layered perovskites.^9^ The use of this “Memory Seed Effect” has led to solar cells with power conversion efficiencies of 17.1% for (C_4_H_9_NH_3_)_2_(CH_3_NH_3_)_3_Pb_4_I_13_.^9^

Organic–inorganic halide perovskites exhibit great compositional flexibility.^4,10^ The A, B, and X sites can be doped in order to fine-tune the electronic structure and band gap for a range of optoelectronic applications.^3,10,11^ In particular, as the electronic structure of organic–inorganic metal halides depends on the B-site cation and halide, halide substitution is an excellent way of tuning the band gap of these materials.^12,13^ For example, halide substitution in FAPbI_3-x_Br_*x*_ (FA = formamidinium) resulted in the band gap being tuned from 1.48 to 2.23 eV.^3^ Unfortunately, photoinduced halide segregation has been reported in CH_3_NH_3_PbI_3–*x*_Br_*x*_, yielding iodine- and bromine-rich domains in the thin films.^14^ Nevertheless, such tunability of the materials is of interest, particularly in the fields of indoor photovoltaics or tandem solar cells, which can utilize two perovskites with different compositions and band gaps.^4^ One method of preventing halide segregation is to use multidentate ligands which create interfaces with a low defect content.^15^

Recently, several new families of layered perovskites have been prepared which incorporate methylammonium in addition to cations which contain aromatic rings such as phenylethylammonium (PEA), 4-aminomethylpyridinium (4AMPY), 3-aminomethylpyridinium (3AMPY), or *meta*-phenylenediammonium (mPDA).^16−18^ Several of these perovskites adopt the Dion–Jacobson structure.^16,17^ Interestingly, the position of the aminomethyl group on the pyridinium ring in 4AMPY and 3AMPY influences the stacking of the inorganic layer containing lead iodide octahedra and results in a decrease in band gap when going from (4AMPY)(CH_3_NH_3_)_*n*-1_Pb_*n*_I_3*n*+1_ to (3AMPY)(CH_3_NH_3_)_*n*-1_Pb_*n*_I_3*n*+1_.^16^ (3AMPY)(CH_3_NH_3_)_3_Pb_4_I_13_ exhibited a power conversion efficiency of 9.20%. In the (mPDA)(CH_3_NH_3_)_*n*-1_Pb_*n*_I_3*n*+1_ system, fabrication of phase pure thin films has been challenging for some values of *n*.^17^ (PEA)_2_(CH_3_NH_3_)_2_Pb_3_I_10_ has been shown to have enhanced moisture stability with respect to CH_3_NH_3_PbI_3_.^18^

The term “zero-dimensional perovskite” has been coined for organic–inorganic metal halides which consist of isolated clusters of metal halide octahedra which are separated by organic cations, although strictly speaking these materials do not possess the perovskite structure. Depending on the nature of the metal and halide, different connectivities of octahedra may exist in the cluster, such as isolated MX_6_ (where M = metal cation and X = halide) or edge-sharing octahedra to make M_2_X_10_ or face-sharing octahedra to make M_2_X_9_. Zero-dimensional materials are already showing interesting properties; for example, (1,3-propanediammonium)_2_Bi_2_I_10_·2H_2_O has been tested as a photodetector, and reproducible photocurrents could be drawn from this material.^19^ (CH_3_NH_3_)_3_Bi_2_I_9_ shows highly anisotropic photoluminescence and has shown evidence of quantum cutting.^20^ In addition, isovalent doping in the zero-dimensional (CH_3_NH_3_)_3_Bi_2–*x*_Sb_*x*_I_9_ has shown a band-bowing phenomena.^21^ Zero-dimensional all-inorganic halides such as A_4_PbX_6_ and Cs_4_SnX_6_ (A = K, Cs, Rb and X = Cl, Br, I) are also of interest, particularly for white light emission, and work by Mohammed et al. has recently found that the local octahedral distortions enabled the formation of self-trapped states.^22^

Motivated by reports of enhanced moisture stability when aromatic organic ammonium cations are used,^18^ along with the huge compositional and structural diversity of organic–inorganic halides, we have explored the synthesis and tunability of new organic–inorganic halides which use the *meta*-xylylenediammonium (MXD, H_3_NCH_2_C_6_H_4_CH_2_NH_3_^2+^) cation.

## Experimental Section

The synthesis of all materials was based on modifications of the method reported by Poglitsch and Weber for the preparation of CH_3_NH_3_PbI_3_.^23^ The appropriate metal oxide was dissolved in 5 mL of HX and heated to 80 °C. Separately, 0.5 mL of HX was added to 0.4 mL *m*-xylylenediamine, resulting in the formation of crystals. These crystals were heated until dissolved. The two solutions were mixed and stirred for 30 min. The solution was cooled to room temperature, and the resulting crystals were filtered. The crystals were dried in a vacuum oven at 80 °C for 2 h.

For the synthesis of MXDBiI_5–*x*_Br_*x*_, 0.47 g Bi_2_O_3_ was added to a mixture of HX acid (X = Br, I). The volumetric ratio of HI:HBr used in the reactions are given in Table 1. The reaction mixture was then placed on a hot plate and heated to 80 °C, with stirring, until all reactants were dissolved. Then, an equimolar quantity of *m*-xylylenediamine (0.14 mL) was added to the solution, and the mixture was stirred for 30 min. After cooling to room temperature, the crystals were filtered off and left to dry in the fume hood.

**Table 1:** Volumetric Ratios of HBr and HI Used in the Synthesis of the MXDBiI_5–*x*_Br_*x*_ Mixed Halide Samples

HI	0.00	0.125	0.250	0.375	0.500	0.625	0.750	0.875	1.000
HBr	1.00	0.875	0.750	0.625	0.500	0.375	0.250	0.125	0.000

Powder X-ray diffraction data were collected on a Panalytical Empyrean Diffractometer using CuK_α1_ radiation in Bragg–Brentano geometry. Data were collected from 5° to 70° with a step size of 0.017° and a time per step of 0.94 s. PXRD data were analyzed using Topas Academic ver. 6.^24^

Single crystal diffraction data were recorded at either 173 or 293 K using a Rigaku FR-X Ultrahigh brilliance Microfocus RA generator/confocal optics and Rigaku XtaLAB P200 diffractometer [Mo Kα radiation (λ = 0.71073 Å)]. Intensity data was collected using ω steps accumulating area detector images spanning at least a hemisphere of reciprocal space (CrystalClear).^25^ The data was processed using CrysAlisPro software.^26^ Structure solution was carried out using SHELXT,^27^ and structure refinement by full matrix least-squares against F^2^ was carried out with SHELXL (2018/3).^28^ Non-hydrogen atoms were refined anisotropically, and carbon-bound hydrogens were refined using a riding model. Ammonium hydrogens were located from the difference Fourier map and refined isotropically subject to distance restraints. Selected crystallographic data are presented in Tables 2 and 3. Deposition numbers 2151572–2151574 contains the supplementary crystallographic data for this paper. These data are provided free of charge by the joint Cambridge Crystallographic Data Centre and Fachinformationszentrum Karlsruhe Access Structures service www.ccdc.cam.ac.uk/structures.

**Table 2:** Refinement Details for MXD_2_PbI_6_, MXDBiI_5_, and MXD_2_Bi_2_Br_12_·2H_2_O

	MXD_2_PbI_6_	MXDBiI_5_	(MXD)_3_Bi_2_Br_12_·2H_2_O
CCDC Code	2151572	2151573	2151574
Formula	C_16_H_28_I_6_N_4_Pb	C_16_H_28_Bi_2_I_10_N_4_	C_24_H_46_Bi_2_Br_12_N_6_O_2_
Formula Weight	1245.01	1963.38	1827.55
Crystal Description	Yellow prism	Red plate	Yellow prism
Crystal Size (mm^3^)	0.12 × 0.05 × 0.02	0.15 × 0.06 × 0.01	0.07 × 0.06 × 0.02
Temperature (K)	173(2)	293(2)	173(2)
Crystal System	Monoclinic	Monoclinic	Triclinic
Space group	*P*2_1_/*c*	*I*2/*m*	*P*1̅
*a* (Å)	10.9539(3)	8.5380(4)	8.0562(2)
*b* (Å)	15.5492(3)	11.6966(6)	14.2053(3)
*c* (Å)	8.8638(2)	19.2708(8)	21.0458(5)
α (deg)			102.611(2)
β (deg)	104.565(3)	99.550(4)	100.779(2)
γ (deg)			99.681(2)
Volume (Å^3^)	1461.20(6)	1897.82(16)	2253.38(9)
Z	2	2	2
ρ (calc, g/cm^3^)	2.830	3.436	2.693
μ (mm^–1^)	12.122	17.407	18.469
F(000)	1104	1696	1668
Reflections collected	18551	6306	39841
Independent reflections (*R*_int_)	3423 (0.0308)	2302 (0.0324)	10230 (0.0393)
Parameters, restraints	148,48	91, 7	481, 33
Goodness-of-fit on F^2^	1.074	1.093	1.091
*R*_1_	0.0179	0.0371	0.0636
*R*_1_ [I > 2σ(I)]	0.0161	0.0278	0.0398
*wR*_2_	0.0356	0.0680	0.0743
*wR*_2_ [I > 2σ(I)]	0.0353	0.0645	0.0696
Largest diff. peak and hole (e/Å^3^)	0.781 and −1.310	0.940 and −1.512	1.706 and −1.458

**Table 3:** M–X Bond Lengths and Octahedral Distortions for MX_6_ Octahedra in MXD_2_PbI_6_, MXDBiI_5_, and MXD_3_Bi_2_Br_12_·2H_2_O

	MXD_2_PbI_6_	MXDBiI_5_	(MXD)_3_Bi_2_Br_12_·2H_2_O	
M-X(1) (Å)	3.20771(17)	2.9710(5)	2.7942(8)	2.9350(8)
M-X(2) (Å)	3.20776(17)	3.0245(6)	2.8029(8)	2.8073(8)
M-X(3) (Å)	3.21169(17)	3.0565(4)	2.9389(8)	2.8203(8)
M-X(4) (Å)	3.21171(17)	3.0565(4)	2.8813(8)	2.8733(8)
M-X(5) (Å)	3.2443(2)	3.2117(6)	2.8836(8)	2.9270(8)
M-X(6) (Å)	3.2443(2)	3.2459(6)	2.8650(8)	2.7722(8)
Bond length distortion (Δ*d,* × 10^–4^)^29^	0.258	10.39	8.65	12.98
Bond angle variance (σ^2^)^29^	31.63	13.13	18.42	7.46

Scanning electron microscopy studies were carried out using a JSM IT200 equipped with a 25 mm^2^ Jeol DrySD EDS detector and a Jeol JSM 5600. UV–vis diffuse reflectance spectra were collected on a Jasco V650 spectrophotometer equipped with an integrating sphere, in the wavelength range 190–900 nm. BaSO_4_ was used as a reference.

## Results and Discussion

### Crystal Structure

The reactions of MXD with either Bi_2_O_3_ and HI or HBr, along with PbO and HI, produced the crystalline products MXDBiI_5_, MXD_3_Bi_2_Br_12_·2H_2_O, and MXD_2_PbI_6_. Their structures were determined from single-crystal X-ray diffraction data, and selected parameters are tabulated in Table 2.

**Figure 1:**
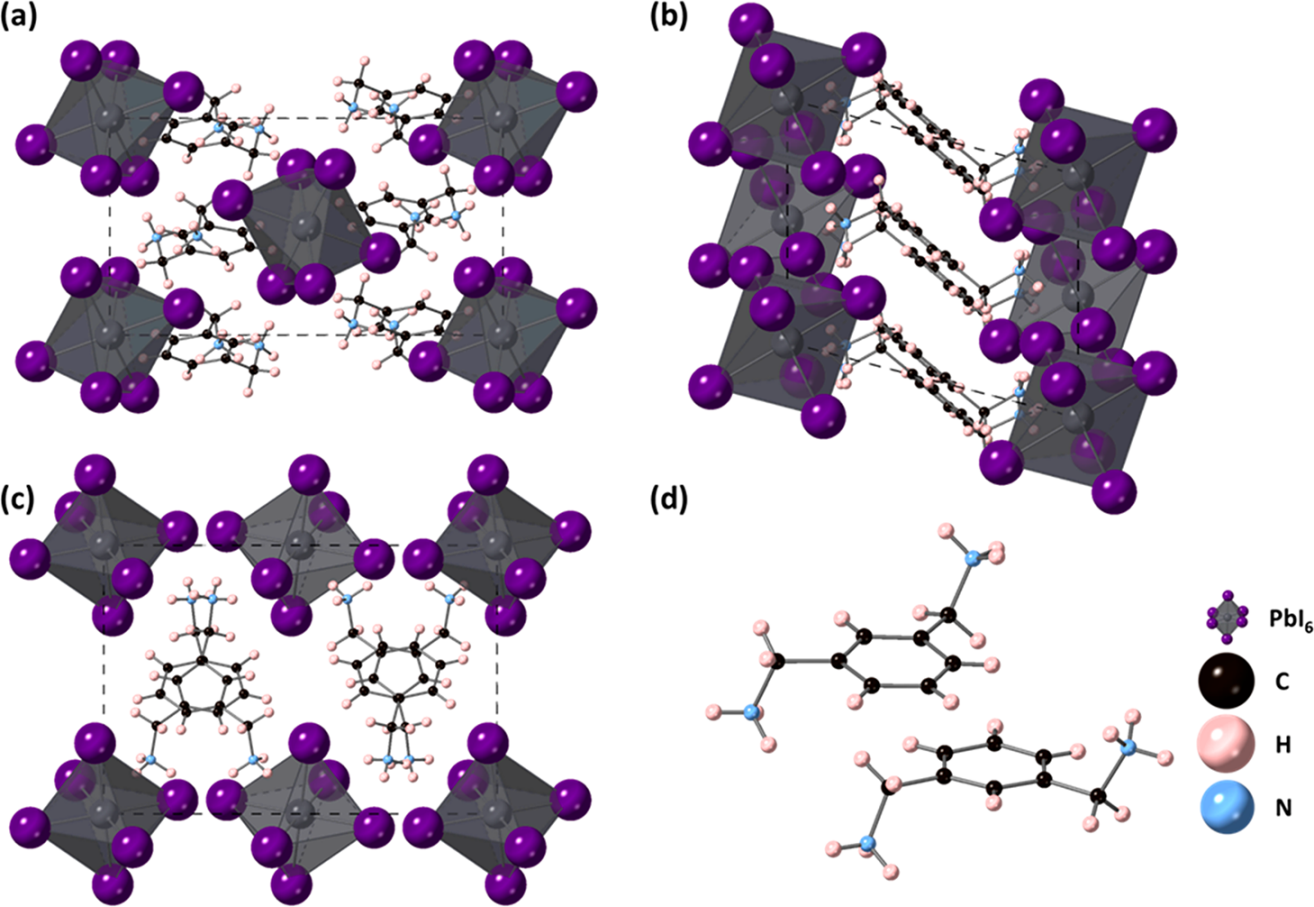
Crystal structure of MXD_2_PbI_6_ (a) viewed down the *a*-axis, (b) viewed down the *b-*axis, (c) viewed down the *c*-axis, and (d) to show the arrangement of adjacent MXD cations.

The structure of MXD_2_PbI_6_ consists of isolated PbI_6_ octahedra separated by MXD cations (Figure 1). Although the structure is not strictly a layered perovskite, the structure can be thought of as a “pseudolayered” material, as the PbI_6_ octahedra form layers in the crystal structure but are too far apart to form the corner-sharing-octahedral connectivity required in the perovskite structure. It is likely that this is driven by the ammonium groups from the large MXD cation, which protrude into the inorganic layer. The Pb–I bond lengths range from 3.20774(17) to 3.2443(2) Å (Table 3). The I–Pb–I bond angles range from 81.808(4)° to 98.192(4)° for *cis* I–Pb–I angles, while the *trans* I–Pb–I are 180°. This deviation away from the ideal octahedral angles indicates that the octahedra show some distortion. Such a distortion can be quantified in terms of the bond angle variance and the bond length distortion, as originally reported by Robinson et al.^29^ Here, we calculate the bond length distortion to be 0.258 and bond angle variance to be 31.63. The bond angle variance here is much smaller than the 234.2 reported for (mPDA)PbI_4_ recently reported.^17^ The aromatic core of the MXD cation is not directly above adjacent MXD cations but is offset. The orientations of the adjacent MXD cations result in NH_3_ groups pointing in three different directions to give a Y-shaped arrangement of the NH_3_ groups, with one pair of NH_3_ groups being almost eclipsed (Figure 1c). Hydrogen bonds between NH_3_ hydrogens and iodine from the PbI_6_ octahedra range from 3.546(2) to 3.718(2) Å. Figure S1 shows the PXRD data of the bulk sample. Pawley fits were carried out using the unit cell parameters and space group obtained from single-crystal diffraction. Twelve Chebyshev background parameters, sample displacement, and profile parameters were also refined. The resulting fit is shown in Figure S1, and this confirms the phase purity of the sample.

**Figure 2:**
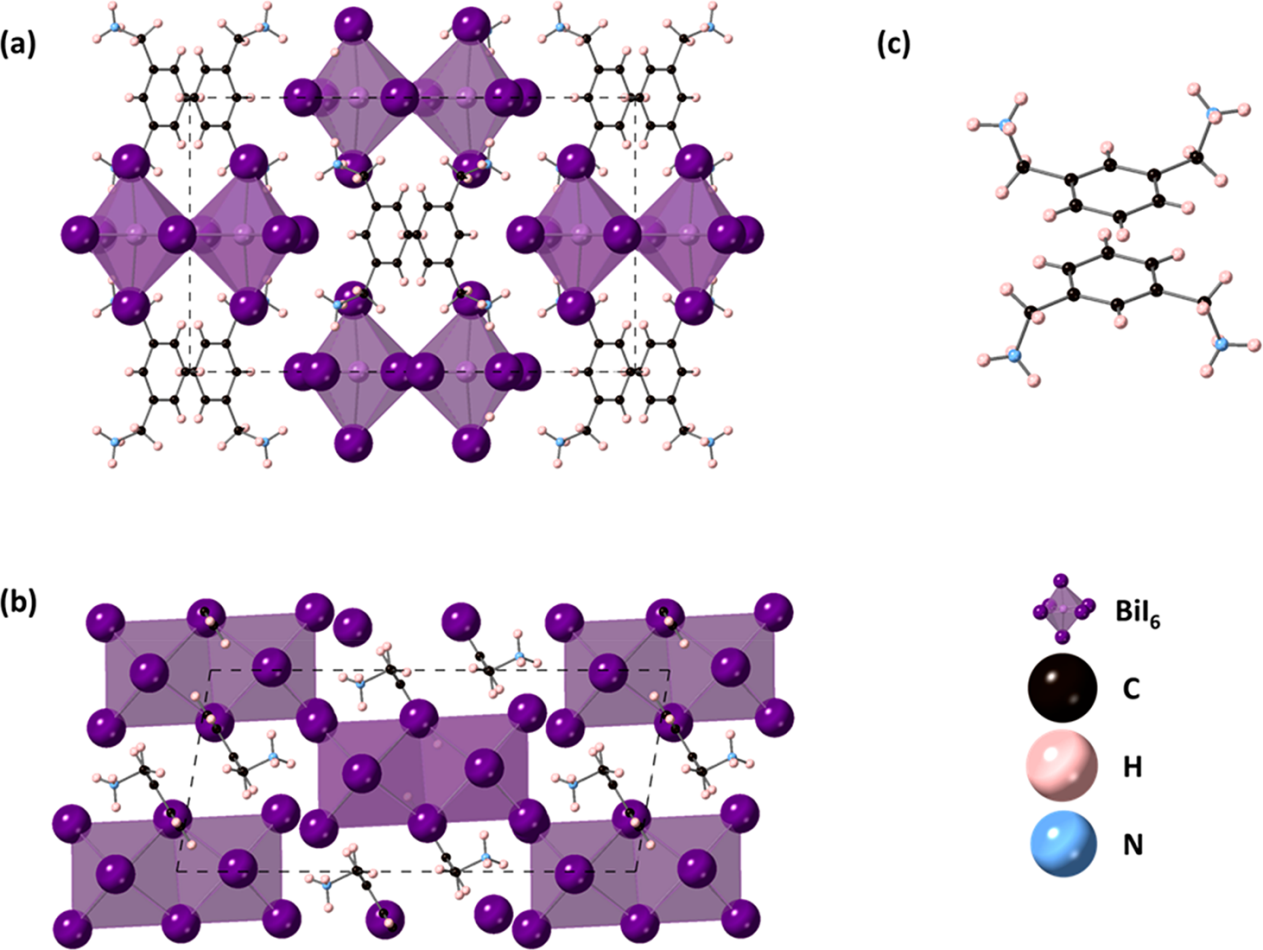
Structure of MXDBiI_5_ (a) viewed down the *a*-axis, (b) viewed down the *b*-axis, and (c) showing the π–π stacking arrangement of the MXD cations.

In contrast to the structure of MXD_2_PbI_6_, which contains isolated PbI_6_ octahedra, the structure of MXDBiI_5_ consists of BiI_6_ octahedra, which share edges to form Bi_2_I_10_ dimers (Figure 2). This kind of structural motif has also been observed in other organic bismuth halides.^30,31^ The Bi_2_I_10_ units are separated by the MXD cations. The Bi–I bond lengths range from 2.9710(5) to 3.2459(6) Å (Table 3). The I–B–I angles range from 171.904(19)° to 178.914(15)° for the iodine in the *trans* positions and from 85.756(15)° to 94.581(17)° in the *cis* position. The Bi octahedra show some distortion, with a bond length distortion of 10.39 and bond angle variance of 13.13. The orientation of the MXD cation plays an important role in holding the structure together. The aromatic rings of adjacent MXD cations are strictly parallel and have a centroid–centroid distance of 3.509(5) Å. The close register of the rings forces the ammonium groups to point to the same side of the ring for each cation, so when considered for each pair of cations, all four NH_3_ groups point in opposite directions. The I1–I4 intercluster distance is 4.1357(6) Å, and this is of particular note, as this is comparable to the short interlayer I–I distances of 4.00 and 4.04 Å, which were reported in (H_3_NC_6_H_4_NH_3_)(CH_3_NH_3_)Pb_2_I_7_ and (H_3_NC_6_H_4_NH_3_)(CH_3_NH_3_)_2_Pb_3_I_10_, respectively.^17^ Although these are thought to be the shortest interlayer iodine–iodine distances in Dion–Jacobson perovskites, we note that in zero-dimensional organic−inorganic halides, short intercluster distances of 3.7253(16) Å have been reported in (H_3_NC_6_H_4_NH_3_)_2_Bi_2_I_10_·4H_2_O, while short interchain distances of 3.871(10) Å have been reported in (H_3_NC_6_H_4_NH_3_)Bi_2_I_8_·I_2_.^17,31,32^ Hydrogen bonds between NH_3_ hydrogens and iodine in the Bi_2_I_10_ units ranged from 3.712(5) to 3.812(6) Å. Figure S2 shows the PXRD data of the bulk MXDBiI_5_ sample. Pawley fits were carried out using the cell parameters and space group obtained from single crystal diffraction MXDBiI_5_ and the same nonstructural parameters that were described for MXD_2_PbI_6_. The resulting fit is shown in Figure S2, and this confirms the phase purity of the sample.

**Figure 3:**
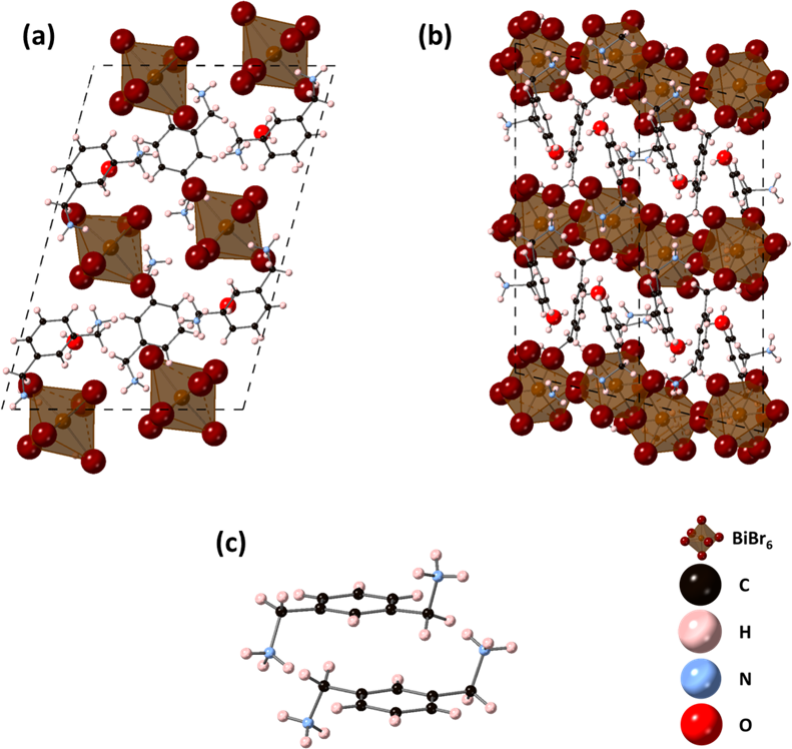
Crystal structure of MXD_3_Bi_2_Br_12_·2H_2_O (a) viewed down the *a*-axis, (b) viewed down the *b*-axis, and (c) arrangement of adjacent MXD cations.

**Figure 4:**
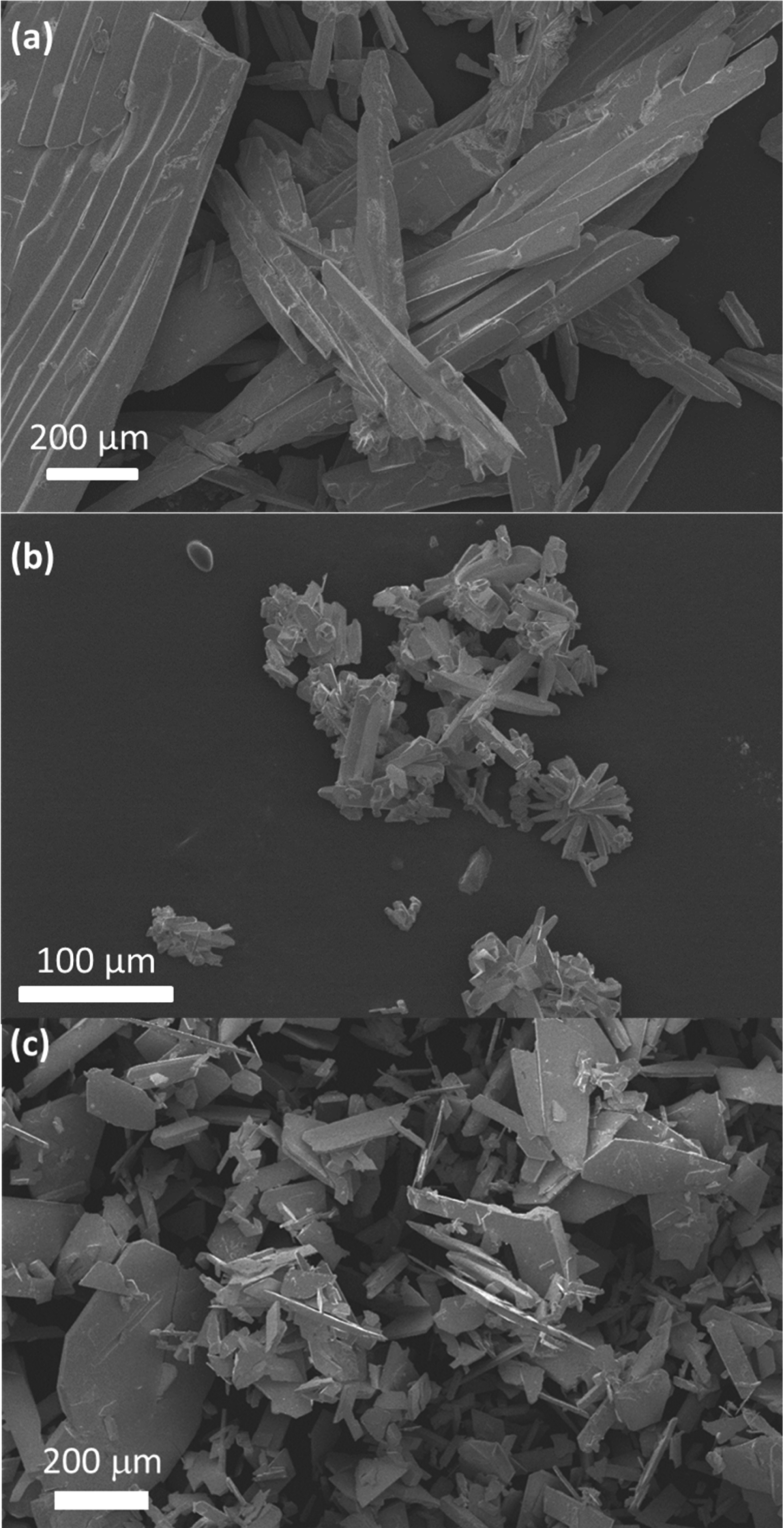
SEM images of (a) MXD_2_PbI_6_, (b) MXDBiI_5_, and (c) MXD_3_Bi_2_Br_12_·2H_2_O.

The structure of MXD_3_Bi_2_Br_12_·2H_2_O consists of isolated BiBr_6_ octahedra, with the asymmetric unit containing two inequivalent BiBr_6_ octahedra (Figure 3). For the Bi(1) octahedron, the Bi–Br bond lengths range from 2.7942(8) to 2.9389(8) Å, while for the Bi(2) octahedron, the Bi–Br bond lengths range from 2.7722(8) to 2.9350(8) Å (Table 3). In addition, the bond angles of Bi(1) range from 84.45(2)° to 99.54(2)° for *cis* bond angles and 170.57(2)° to 176.29(3)° for *trans* angles, while for Bi(2), the bond angles range from 174.21(2)° to 177.20(3)° for *trans* bond angles and 85.46(2)° to 95.13(2)° for *cis* bond angles. Calculation of the bond length distortion and bond angle variance show that the BiBr_6_ octahedra exhibit different levels of distortion, with Bi(1) showing the greater angular variance than the Bi(2), while Bi(1) shows less distortion of bond lengths than Bi(2) (Table 3). The bond angle variance of the BiBr_6_ octahedra are much smaller than that of MXD_2_PbI_6_, which also contains isolated octahedra (PbI_6_) in the structure. They are also significantly smaller than the bond angle variance reported for (mPDA)PbI_4_, but are comparable to those reported for some quinoline and isoquinoline lead halides.^17,33^ The shortest hydrogen bonds are formed between the NH_3_ and H_2_O, with N···O distances of 2.757(12) Å and 2.822(10) Å. Figure S3 shows the PXRD data of the bulk MXD_3_Bi_2_Br_12_·2H_2_O sample. Pawley fits were carried out using the cell parameters and space group obtained from single crystal diffraction and parameters described for MXD_2_PbI_6_. The resulting fit is shown in Figure S3, and this confirms the phase purity of the sample. SEM images of MXD_2_PbI_6_, MXDBiI_5_, and MXD_3_Bi_2_Br_12_·2H_2_O are shown in Figure 4.

SEM images of MXD_2_PbI_6_, MXDBiI_5_, and MXD_3_Bi_2_Br_12_·2H_2_O are shown in Figure 4. MXD_2_PbI_6_ displays aggregates of crystals, with a needle-like morphology, of dimensions of approximately 600 μm by 40 μm. Crystallites of MXDBiI_5_ also show a needle-like morphology, although the crystals are much shorter than those reported for the MXD_2_PbI_6_, with dimensions of approximately 50–75 μm by 20 μm. The MXD_3_Bi_2_Br_12_·2H_2_O sample consists of much thinner crystallites with a narrow, plate-like morphology. A range of crystallite sizes can be observed, with typical dimensions being around 50 μm by 100–200 μm. Although the control of morphology was not considered in this study, this will be of interest in the future, when manufacturing these materials into thin films, as any pinholes in thin films as a result of surface morphology can influence factors such as device performance in photovoltaics or LEDs.

### Halide Substitution

**Figure 5:**
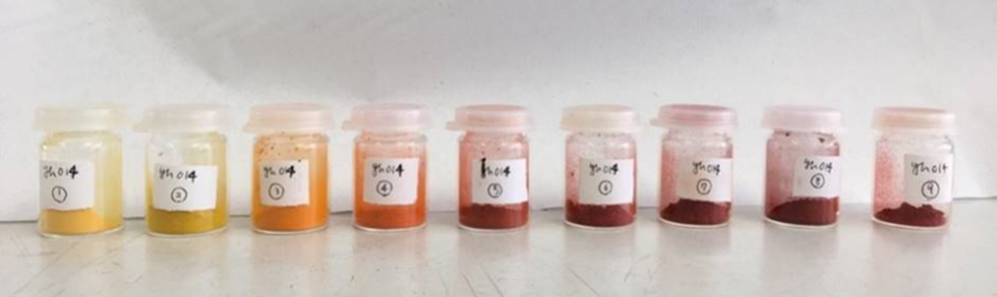
Photograph of halide substituted samples, MXDBiI_5–*x*_Br_*x*_.

It is well-known that the valence band and conduction bands in organic–inorganic halides comprise orbital contributions from both the halide and the inorganic cation.^34^ Therefore, isovalent doping on the anion site, e.g., replacing I^–^ with Br^–^, is a particularly useful technique to tune the band gap of the CH_3_NH_3_PbI_3_ perovskites.^13,35^ This is often termed halide substitution and results in the creation of materials with compositions such as CH_3_NH_3_PbI_3–*x*_Br_*x*_. Zero-dimensional inorganic–organic halides commonly exhibit a similar electronic structure to the 3D analogues, but with greater orbital decoupling;^36^ therefore, we decided to probe halide substitution in MXDBiI_5–*x*_Br_*x*_, in order to determine the doping limit of Br^–^ in the MXDBiI_5_ structure type. A photograph of the MXDBiI_5–*x*_Br_*x*_ samples is shown in Figure 5 and shows that the color of the samples can be varied from yellow (Br-rich samples) to dark red (I-rich samples). As these samples were synthesized using solution based-routes, the ratio of I to Br was determined using energy dispersive spectroscopy (EDS) using the SEM. The I:Br ratio in a sample is not the same as the I:Br ratio in its precursor solutions, but across the I:Br ratios studied, the ratio in the sample can be related directly to its ratio in solution (Figure S5).^36^ We note that similar phenomena have been reported when two different cations are used in the synthesis of Ruddlesden–Popper phases, as nonstoichiometric ratios of reagents must be used to isolate the phase pure product.^37^ In addition, in the synthesis of Cs_2_SnX_6_ (X = Cl, Br, I) mixed halides, the products were found to be richer in the Cl or Br than would be expected given the ratios in the precursors, and this has been attributed to the difference in solubility of halides in solution.^38^

**Figure 6:**
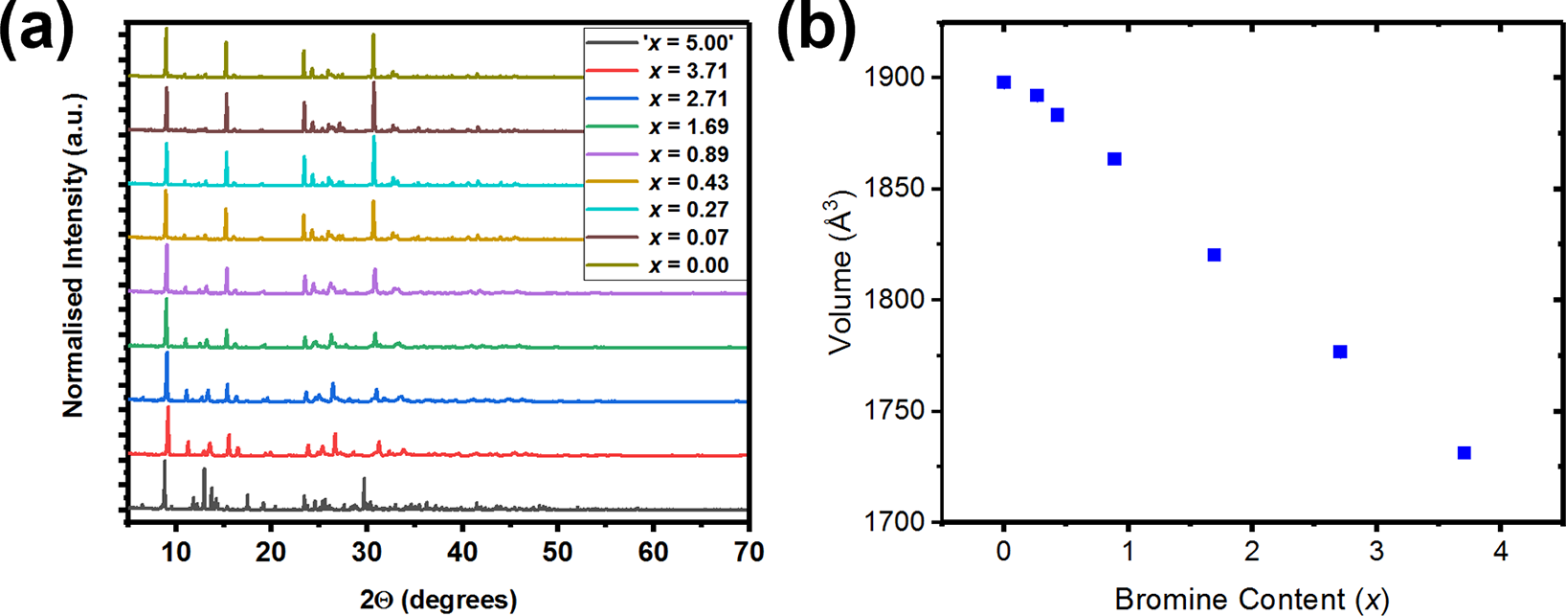
(a) PXRD data of MXDBiI_5–*x*_Br_*x*_ and (b) variation of unit cell volume with bromine content for MXDBiI_5–*x*_Br_*x*_. The bromine-end member (*x* = 5) has a different structure type so has been excluded from part b.

The PXRD data of MXDBiI_5–*x*_Br_*x*_ samples are shown in Figure 6, and a representative SEM image of the polycrystalline sample of MXDBiI_4.11_Br_0.89_ is shown in Figure S5. As can be seen, the 100% Br sample (i.e., “*x* = 5”) exhibits a completely different PXRD pattern to the other samples prepared in this series, which indicates that it adopts a completely different structure type, MXD_3_Bi_2_Br_12_·2H_2_O (vide supra). However, with only a small amount of HI in the precursor solution (see Table 1 and Figure S4), the MXDBiI_5_ structure type is adopted, and all peaks can be indexed to a monoclinic unit cell in space group *I*2/*m*. This structure type is adopted for all MXDBiI_5–*x*_Br_*x*_ compositions which have a bromine content, *x*, between 0 and 3.71 (i.e., having iodine contents from 1.29 up to 5.0). In order to determine the extent of the solid solution in MXDBiI_5–*x*_Br_*x*_, Pawley refinements were carried out. During the refinements, 12 Chebyshev polynomial terms were used to fit the background, and in addition, unit cell parameters, profile parameters, and specimen displacement were all refined. The resulting variation of unit cell volume with iodine content is shown in Figure 6b, and the corresponding unit cell parameters are plotted in the Supporting Information (Figure S6). As can be seen from Figure 6b, there is a linear relationship between bromine content (*x*) and unit cell volume, in agreement with Vegard’s law, with the exception of a slight leveling off at the lowest bromine contents. This shows that there is a large region of solid solution in the MXDBiI_5–*x*_Br_*x*_ system. This region of solid solution ranges from a bromine content, *x*, of 0 to 3.71, and it is likely that a full solid solution could be obtained with further optimization of synthetic conditions. As the bromine content increases, the β angle increases, but there is a leveling off at the highest iodine contents (which corresponds to the lowest bromine contents). The *a*, *b*, and *c* unit cell parameters all show a linear relationship with bromine content and exhibit a smaller leveling off at the lowest bromine contents. The decrease in unit cell parameters with increasing bromine content is expected due to the smaller size of Br^–^ (1.96 Å) with respect to I^–^ (2.20 Å).^39^

**Figure 7:**
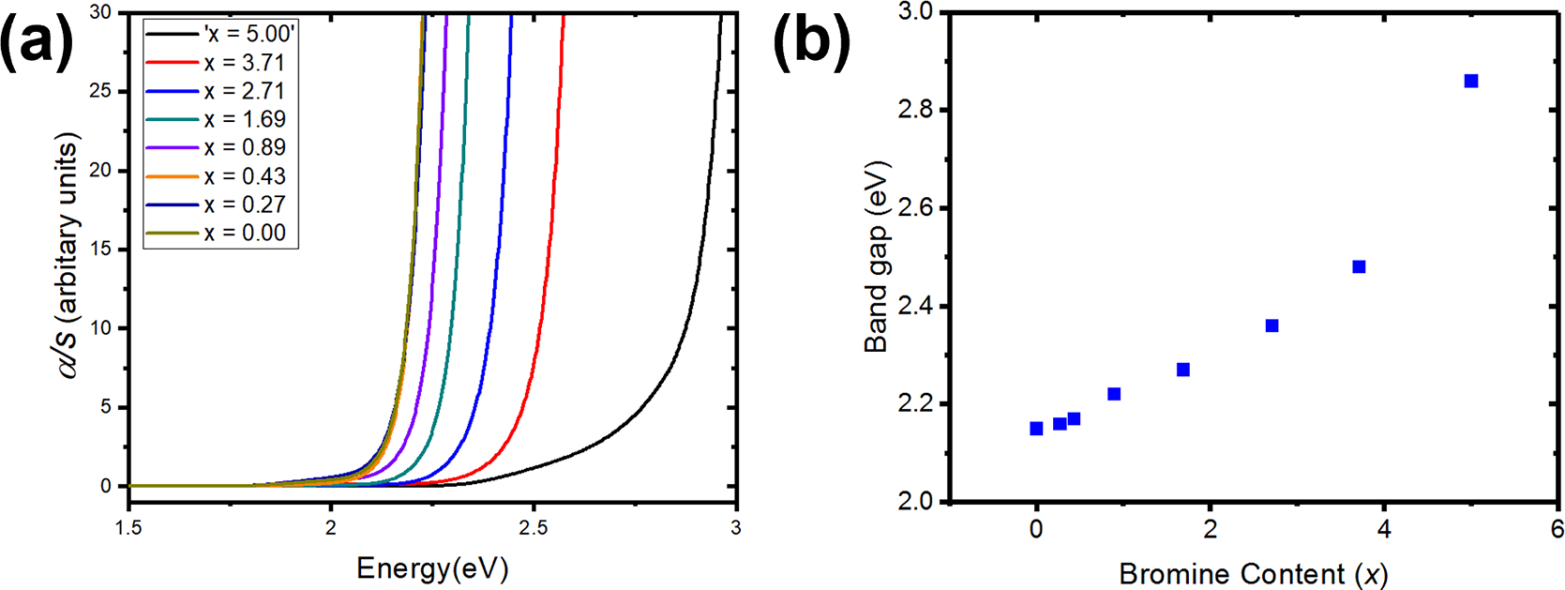
(a) UV–vis diffuse reflectance spectra for MXDBiI_5*-x*_Br_*x*_ converted using the Kubelka–Munk function (α/*s* = (1 – *R*)^2^/2*R*) and (b) the variation of band gap with bromine content for MXDBiI_5–*x*_Br_*x*_. The *x* = 5 sample has a different structure type.

The Kubelka–Munk transformations of the UV–vis diffuse reflectance spectra of the MXDBiI_5–*x*_Br_*x*_ samples and MXD_3_Bi_2_Br_12_·2H_2_O are shown in Figure 7, while the MXD_2_PbI_6_ spectrum is shown in Figure S7. Two absorption features are observed in the spectra for MXDBiI_5–*x*_Br_*x*_. For MXDBiI_5–*x*_Br_*x*_, the first feature is in the 2.36–2.06 eV range (corresponding to 525–600 nm) and the second feature is centered around 1.9 eV (∼650 nm), while for MXD_2_PbI_6_, the peak occurs at ∼500 nm (2.5 eV) We note that dual features have been observed in both UV–vis spectroscopy and photoluminescence measurements for both 2D, layered materials, and zero-dimensional materials.^40−42^ For example, Nag et al. noticed dual-emission in photoluminescence studies of single crystals of (PEA)_2_SnI_4_ (PEA = phenylethylammonium), which was also accompanied by two absorption features in UV–vis absorption spectra of the same material.^41^ A similar behavior was also observed for (4-AMP)SnI_4_ (4-AMP = (4-aminomethyl)piperidinium).^41^ Dual PL emission has also been observed for the zero-dimensional TPA_2_SbCl_5_ (TPA = tetrapropylammonium).^42^ To date, the presence of dual features in the photoluminescence or absorption spectra has been attributed to differences in the edge or bulk of the crystals or self-trapped excitons.^41,42^ As the bromide content is increased in MXDBiI_5–*x*_Br_*x*_, the absorption edge shifts to larger energies, which is in agreement with an increase in band gap with increasing Br content. The resulting band gaps were estimated using Tauc plots and are listed in Table 4. The lowest band gaps were obtained for the iodine-rich compositions, with MXDBiI_5_ exhibiting the lowest band gap in the series (2.15 eV) and MXD_3_Bi_2_Br_12_·2H_2_O the largest (2.86 eV). In contrast, the band gap of MXD_2_PbI_6_ was determined to be 2.33 eV. The variation of band gap with bromine content is shown in Figure 7b and shows an increase in band gap with increasing bromine content, although this trend is not linear. We also note that the band gap of MXDBiI_5–*x*_Br_*x*_ can be tuned over a similar range to that observed for the FAPbI_3–*x*_Br_*x*_ perovskites, which allowed tuning of the band gap from 1.48 to 2.25 eV.^3^ The high crystallinity of these samples warrants further investigation into the photostability of MXDBiI_5-x_Br_*x*_, as in the mixed cation, mixed halide perovskites, Cs_*y*_FA_1–*y*_PbI_3–*x*_Br_*x*_, a high level of crystallinity was found to suppress halide segregation.^43^

**Table 4:** Bromine Content in MXDBiI_5–*x*_Br_*x*_ (Determined from EDS) and Corresponding Band Gap of MXDBiI_5–*x*_Br_*x*_ Samples

Bromine Content (*x*) in MXDBiI_5-*x*_Br_*x*_	Band Gap (eV)
0.00	2.15
0.27	2.16
0.43	2.17
0.89	2.22
1.69	2.27
2.71	2.36
3.71	2.48
MXD_3_Bi_2_Br_12_·2H_2_O (*x* = 5)	2.86

## Conclusions

Here, we have reported the synthesis and characterization of three new organic–inorganic metal halides, using the *meta*-xylylenediammonium (MXD) cation: MXDBiI_5_, MXD_3_Bi_2_Br_12_·2H_2_O, and MXD_2_PbI_6_. MXDBiI_5_ has short intercluster I–I distances and π–π stacking between the MXD cations, while also possessing the lowest band gap of the materials studied here. We explored the tunability of MXDBiI_5_ through halide substitution and found that a large region of solid solution exists in the MXDBiI_5–*x*_Br_*x*_ system (where *x* = 0 to 3.71). This work highlights the fact that zero-dimensional organic–inorganic halides are also highly tunable semiconductors and opens up the way for further studies of the physics and the long-term stability of these materials.

## Supporting Information Available

PXRD data, SEM images, graphs of unit cell parameters, UV–vis spectra (PDF)

## Accession Codes

CCDC 2151572–2151574 contain the supplementary crystallographic data for this paper. These data can be obtained free of charge via www.ccdc.cam.ac.uk/data_request/cif, or by emailing data_request@ccdc.cam.ac.uk, or by contacting The Cambridge Crystallographic Data Centre, 12 Union Road, Cambridge CB2 1EZ, UK; fax: +44 1223 336033.

## Notes

Supporting data available at https://doi.org/10.17630/956ff492-e63c-4a36-b9a7-6cbfec1a0b3c.

## References

[ref1] Kojima A.; Teshima K.; Shirai Y.; Miyasaka T. Organometal Halide Perovskites as Visible-Light Sensitizers for Photovoltaic Cells. J. Am. Chem. Soc. 2009, 131 (17), 6050–6051. 10.1021/ja809598r. 19366264

[ref2] Lee M. M.; Teuscher J.; Miyasaka T.; Murakami T. N.; Snaith H. J. Efficient Hybrid Solar Cells Based on Meso-Superstructured Organometal Halide Perovskites. Science 2012, 338 (6107), 643–647. 10.1126/science.1228604. 23042296

[ref3] Eperon G. E.; Stranks S. D.; Menelaou C.; Johnston M. B.; Herz L. M.; Snaith H. J. Formamidinium lead trihalide: a broadly tunable perovskite for efficient planar heterojunction solar cells. Energy Environ. Sci. 2014, 7 (3), 982–988. 10.1039/c3ee43822h.

[ref4] McMeekin D. P.; Sadoughi G.; Rehman W.; Eperon G. E.; Saliba M.; Hoerantner M. T.; Haghighirad A.; Sakai N.; Korte L.; Rech B.; et al. A mixed-cation lead mixed-halide perovskite absorber for tandem solar cells. Science 2016, 351 (6269), 151–155. 10.1126/science.aad5845. 26744401

[ref5] https://www.nrel.gov/pv/cell-efficiency.html. (accessed 23rd December 2021).

[ref6] Whitfield P. S.; Herron N.; Guise W. E.; Page K.; Cheng Y. Q.; Milas I.; Crawford M. K. Structures, Phase Transitions and Tricritical Behavior of the Hybrid Perovskite Methyl Ammonium Lead Iodide. Sci. Rep. 2016, 6, 35685 10.1038/srep35685. 27767049 PMC5073364

[ref7] Saparov B.; Mitzi D. B. Organic-Inorganic Perovskites: Structural Versatility for Functional Materials Design. Chem. Rev. 2016, 116 (7), 4558–4596. 10.1021/acs.chemrev.5b00715. 27040120

[ref8] Tsai H. H.; Nie W. Y.; Blancon J. C.; Toumpos C. C. S.; Asadpour R.; Harutyunyan B.; Neukirch A. J.; Verduzco R.; Crochet J. J.; Tretiak S.; et al. High-efficiency two-dimensional Ruddlesden-Popper perovskite solar cells. Nature 2016, 536 (7616), 312–316. 10.1038/nature18306. 27383783

[ref9] Sidhik S.; Li W. B.; Samani M. H. K.; Zhang H.; Wang Y. F.; Hoffman J.; Fehr A. K.; Wong M. S.; Katan C.; Even J.; et al. Memory Seeds Enable High Structural Phase Purity in 2D Perovskite Films for High-Efficiency Devices. Adv. Mater. 2021, 33, 2007176 10.1002/adma.202007176. 34096115

[ref10] Jesper Jacobsson T.; Correa-Baena J.-P.; Pazoki M.; Saliba M.; Schenk K.; Gratzel M.; Hagfeldt A. Exploration of the compositional space for mixed lead halogen perovskites for high efficiency solar cells. Energy Environ. Sci. 2016, 9 (5), 1706–1724. 10.1039/C6EE00030D.

[ref11] Hao F.; Stoumpos C. C.; Chang R. P. H.; Kanatzidis M. G. Anomalous Band Gap Behavior in Mixed Sn and Pb Perovskites Enables Broadening of Absorption Spectrum in Solar Cells. J. Am. Chem. Soc. 2014, 136 (22), 8094–8099. 10.1021/ja5033259. 24823301

[ref12] Li C.; Wei J.; Sato M.; Koike H.; Xie Z. Z.; Li Y. Q.; Kanai K.; Kera S.; Ueno N.; Tang J. X. Halide-Substituted Electronic Properties of Organometal Halide Perovskite Films: Direct and Inverse Photoemission Studies. ACS Appl. Mater. Int. 2016, 8 (18), 11526–11531. 10.1021/acsami.6b02692. 27101940

[ref13] Zarick H. F.; Soetan N.; Erwin W. R.; Bardhan R. Mixed halide hybrid perovskites: a paradigm shift in photovoltaics. J. Mater. Chem. A 2018, 6 (14), 5507–5537. 10.1039/C7TA09122B.

[ref14] Hoke E. T.; Slotcavage D. J.; Dohner E. R.; Bowring A. R.; Karunadasa H. I.; McGehee M. D. Reversible photo-induced trap formation in mixed-halide hybrid perovskites for photovoltaics. Chem. Sci. 2015, 6 (1), 613–617. 10.1039/C4SC03141E. 28706629 PMC5491962

[ref15] Hassan Y.; Park J. H.; Crawford M. L.; Sadhanala A.; Lee J.; Sadighian J. C.; Mosconi E.; Shivanna R.; Radicchi E.; Jeong M.; et al. Ligand-engineered bandgap stability in mixed-halide perovskite LEDs. Nature 2021, 591 (7848), 72–77. 10.1038/s41586-021-03217-8. 33658694

[ref16] Li X. T.; Ke W. J.; Traore B.; Guo P. J.; Hadar I.; Kepenekian M.; Even J.; Katan C.; Stoumpos C. C.; Schaller R. D.; et al. Two-Dimensional Dion-Jacobson Hybrid Lead Iodide Perovskites with Aromatic Diammonium Cations. J. Am. Chem. Soc. 2019, 141 (32), 12880–12890. 10.1021/jacs.9b06398. 31313919

[ref17] Gao L.; Li X.; Traore B.; Zhang Y.; Fang J.; Yu H.; Even J.; Katan C.; Zhao K.; Liu S.; et al. m-Phenylenediammonium as a New Spacer for Dion–Jacobson Two-Dimensional Perovskites. J. Am. Chem. Soc. 2021, 143 (31), 12063–12073. 10.1021/jacs.1c03687. 34342223

[ref18] Smith I. C.; Hoke E. T.; Solis-Ibarra D.; McGehee M. D.; Karunadasa H. I. A Layered Hybrid Perovskite Solar-Cell Absorber with Enhanced Moisture Stability. Angew. Chem., Int. Ed. 2014, 53 (42), 11232–11235. 10.1002/anie.201406466. 25196933

[ref19] Pious J. K.; Katre A.; Muthu C.; Chakraborty S.; Krishna S.; Nair V. C. Zero-Dimensional Lead-Free Hybrid Perovskite-like Material with a Quantum-Well Structure. Chem. Mater. 2019, 31 (6), 1941–1945. 10.1021/acs.chemmater.8b04642.

[ref20] Ni C. S.; Hedley G.; Payne J.; Svrcek V.; McDonald C.; Jagadamma L. K.; Edwards P.; Martin R.; Jain G.; Carolan D.; et al. Charge carrier localised in zero-dimensional (CH_3_NH_3_)_3_Bi_2_I_9_ clusters. Nat. Commun. 2017, 8, 170 10.1038/s41467-017-00261-9. 28761100 PMC5537240

[ref21] Chatterjee S.; Payne J.; Irvine J. T. S.; Pal A. J. Bandgap bowing in a zero-dimensional hybrid halide perovskite derivative: spin-orbit coupling versus lattice strain. J. Mater. Chem. A 2020, 8 (8), 4416–4427. 10.1039/C9TA12263J.

[ref22] Yin J.; Bredas J. L.; Bakr O. M.; Mohammed O. F. Boosting Self-Trapped Emissions in Zero-Dimensional Perovskite Heterostructures. Chem. Mater. 2020, 32 (12), 5036–5043. 10.1021/acs.chemmater.0c00658.

[ref23] Poglitsch A.; Weber D. Dynamic Disorder in Methylammoniumtrihalogenoplumbates (II) Observed by Millimeter-Wave Spectroscopy. J. Chem. Phys. 1987, 87 (11), 6373–6378. 10.1063/1.453467.

[ref24] Coelho A. A. TOPAS and TOPAS-Academic: an optimization program integrating computer algebra and crystallographic objects written in C plus. J. Appl. Crystallogr. 2018, 51, 210–218. 10.1107/S1600576718000183.

[ref25] CrystalClear-SM Expert ver. 2.1; Rigaku Americas, The Woodlands, Texas, USA and Rigaku Corporation, Tokyo, Japan, 2015.

[ref26] CrysAlisPro ver. 1.171.38.46, ver. 1.171.41.93a, and ver. 1.171.41.122a; Rigaku Oxford Diffraction, Rigaku Corporation, Oxford, U.K., 2015, 2020, and 2021.

[ref27] Sheldrick G. M. SHELXT - Integrated space-group and crystal-structure determination. Acta Crystallogr., Sect. A: Found. Adv. 2015, 71, 3–8. 10.1107/S2053273314026370. 25537383 PMC4283466

[ref28] Sheldrick G. M. Crystal structure refinement with SHELXL. Acta Crystallogr., Sect. C: Struct. Chem. 2015, 71, 3–8. 10.1107/S2053229614024218. 25567568 PMC4294323

[ref29] Robinson K.; Gibbs G. V.; Ribbe P. H. Quadriatic Elongation- Quantitative Measure of Distortion in Coordination Polyhedra. Science 1971, 172 (3983), 567–570. 10.1126/science.172.3983.567. 17802221

[ref30] Hrizi C.; Trigui A.; Abid Y.; Chniba-Boudjada N.; Bordet P.; Chaabouni S. alpha- to beta- (C_6_H_4_(NH_3_)_2_)_2_Bi_2_I_10_ reversible solid-state transition, thermochromic and optical studies in the p-phenylenediamine-based iodobismuthate(III) material. J. Solid State Chem. 2011, 184 (12), 3336–3344. 10.1016/j.jssc.2011.10.004.

[ref31] Hrizi C.; Samet A.; Abid Y.; Chaabouni S.; Fliyou M.; Koumina A. Crystal structure, vibrational and optical properties of a new self-organized material containing iodide anions of bismuth(III), (C_6_H_4_(NH_3_)_2_)_2_Bi_2_I_10_ center dot 4H_2_O. J. Mol. Struct. 2011, 992 (1–3), 96–101. 10.1016/j.molstruc.2011.02.051.

[ref32] Shestimerova T. A.; Golubev N. A.; Yelavik N. A.; Bykov M. A.; Grigorieva A. V.; Wei Z.; Dikarev E. V.; Shevelkov A. V. Role of I_2_ Molecules and Weak Interactions in Supramolecular Assembling of Pseudo-Three-Dimensional Hybrid Bismuth Polyiodides: Synthesis, Structure, and Optical Properties of Phenylenediammonium Polyiodobismuthate(III). Cryst. Growth Des. 2018, 18 (4), 2572–2578. 10.1021/acs.cgd.8b00179.

[ref33] Guo Y. Y.; Lightfoot P. Structural diversity of lead halide chain compounds, APbX_3_, templated by isomeric molecular cations. Dalton Trans 2020, 49 (36), 12767–12775. 10.1039/D0DT02782K. 32959845

[ref34] Umari P.; Mosconi E.; De Angelis F. Relativistic GW calculations on CH_3_NH_3_PbI_3_ and CH_3_NH_3_SnI_3_ Perovskites for Solar Cell Applications. Sci. Rep. 2015, 4, 4467 10.1038/srep04467. PMC5394751 24667758

[ref35] Mosconi E.; Amat A.; Nazeeruddin M. K.; Gratzel M.; De Angelis F. First-Principles Modeling of Mixed Halide Organometal Perovskites for Photovoltaic Applications. J. Phys. Chem. C 2013, 117 (27), 13902–13913. 10.1021/jp4048659.

[ref36] Yin J.; Maity P.; De Bastiani M.; Dursun I.; Bakr O. M.; Bredas J. L.; Mohammed O. F. Molecular behavior of zero-dimensional perovskites. Sci. Adv. 2017, 3 (12), 1701793 10.1126/sciadv.1701793. PMC5731998 29250600

[ref37] Stoumpos C. C.; Cao D. H.; Clark D. J.; Young J.; Rondinelli J. M.; Jang J. I.; Hupp J. T.; Kanatzidis M. G. Ruddlesden-Popper Hybrid Lead Iodide Perovskite 2D Homologous Semiconductors. Chem. Mater. 2016, 28, 2852–2867. 10.1021/acs.chemmater.6b00847.

[ref38] Karim M. M. S; Ganose A. M.; Pieters L.; Leung W. W. W.; Wade J.; Zhang L.; Scanlon D. O.; Palgrave R. G. Anion Distribution, Structural Distortion, and Symmetry-Driven Optical Band Gap Bowing in Mixed Halide Cs_2_SnX_6_ Vacancy Ordered Double Perovskites. Chem. Mater. 2019, 31 (22), 9430–9444. 10.1021/acs.chemmater.9b03267. 32116409 PMC7046317

[ref39] Shannon R. D. Revised effective ionic radii and systematic studies of interatomic distances in halides and chalcogenides. Acta Crystallogr., Sect. A: Found. Adv. 1976, 32, 751–767. 10.1107/S0567739476001551.

[ref40] Sheikh T.; Shinde A.; Mahamuni S.; Nag A. Possible Dual Bandgap in (C_4_H_9_NH_3_)_2_PbI_4_ 2D Layered Perovskite: Single-Crystal and Exfoliated Few-Layer. ACS Energy Lett. 2018, 3 (12), 2940–2946. 10.1021/acsenergylett.8b01799.

[ref41] Nawale V. V.; Sheikh T.; Nag A. Dual Excitonic Emission in Hybrid 2D Layered Tin Iodide Perovskites. J. Phys. Chem. C 2020, 124 (38), 21129–21136. 10.1021/acs.jpcc.0c05301.

[ref42] Peng H.; Tian Y.; Wang X. X.; Huang T.; Xiao Y. H.; Dong T. T.; Hu J. M.; Wang J. P.; Zou B. S. Bulk assembly of a 0D organic antimony chloride hybrid with highly efficient orange dual emission by self-trapped states. J. Mater. Chem. C 2021, 9 (36), 12184–12190. 10.1039/D1TC02906A.

[ref43] Rehman W.; McMeekin D. P.; Patel J. B.; Milot R. L.; Johnston M. B.; Snaith H. J.; Herz L. M. Photovoltaic mixed-cation lead mixed-halide perovskites: links between crystallinity, photo-stability and electronic properties. Energy Environ. Sci. 2017, 10 (1), 361–369. 10.1039/C6EE03014A.

