# Supplementary material for: Synthesis, Structure, and Tunability of Zero-Dimensional Organic–Inorganic Metal Halides Utilizing the m-Xylylenediammonium Cation: MXD2PbI6, MXDBiI5, and MXD3Bi2Br12·2H2O
Source: Cryst Growth Des. 2022 Apr 28;22(6):3815–23. doi: 10.1021/acs.cgd.2c00187 (PMC9490867; doi:10.1021/acs.cgd.2c00187)
Supplement: Supplementary file 1 — cg2c00187_si_001.pdf [file cg2c00187_si_001.pdf]

Supplementary Information for

Synthesis, Structure and Tunability of Zero Dimensional Organic-Inorganic Metal Halides Utilising the m-Xylylenediammonium Cation: MXD_2PbI_6, $MXDBiI_5$ and $MXD_3Bi_2Br_{12}\cdot 2H_2O$

Pia S. Klee[1], Yuri Hirano[1], David B. Cordes[1], Alexandra M. Z. Slawin[1], Julia L. Payne[1*]

[1]School of Chemistry, University of St Andrews, North Haugh, St Andrews, Fife. KY16 9ST.

**jlp8@st-andrews.ac.uk*

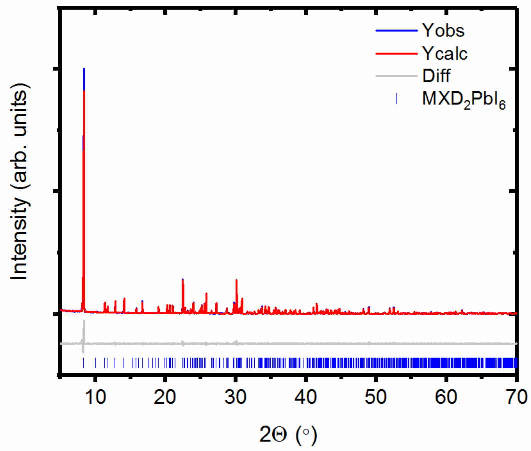

Figure S1: Pawley fit to data obtained for a bulk sample of MXD_2PbI_6.

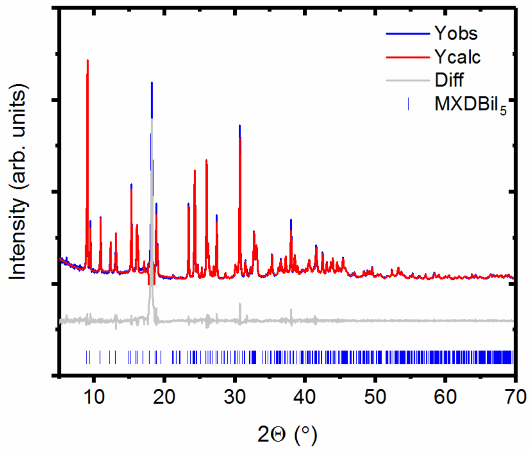

Figure S2: Pawley fit to PXRD data obtained for a bulk sample of $MXDBiI_5$. The peak at 18.2° has been excluded from the refinement as it comes from the Teflon used in the sample holder.

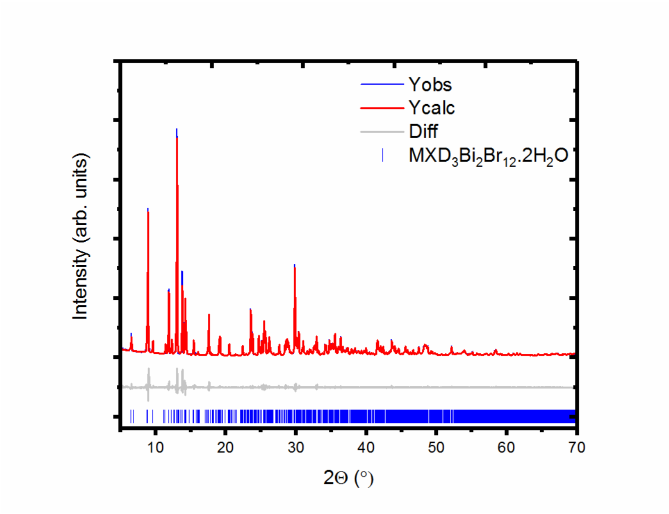

Figure S3: Pawley fit to the PXRD data obtained for a bulk sample of $MXD_3Bi_2Br_{12}{\cdot}2H_2O$.

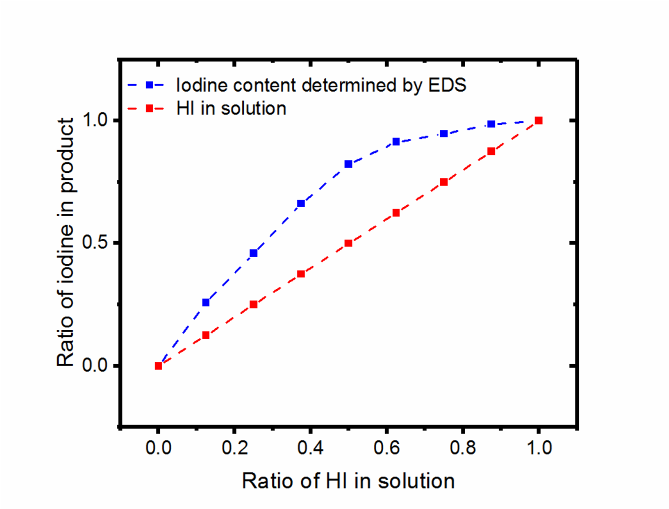

Figure S4: Relationship between ratio of iodine in product (determined using EDS) and ratio of HI in precursor solution. The iodine content in the product can be expressed through the following equation: $y = -1.2302x^2 + 2.2184x - 0.0015$. We note that we have not tested the validity of this expression to other halide perovskites that can be prepared in this way.

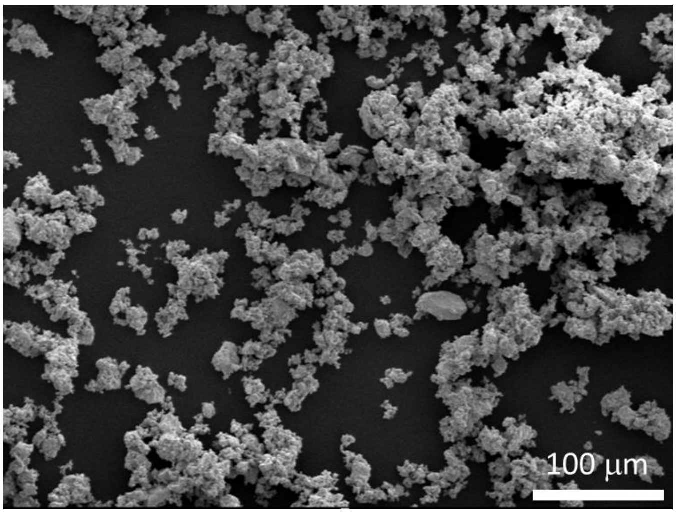

Figure S5: SEM image of a typical polycrystalline sample of $MXDBiI_{5-x}Br_x$ ($MXDBiI_{4.11}Br_{0.89}$)

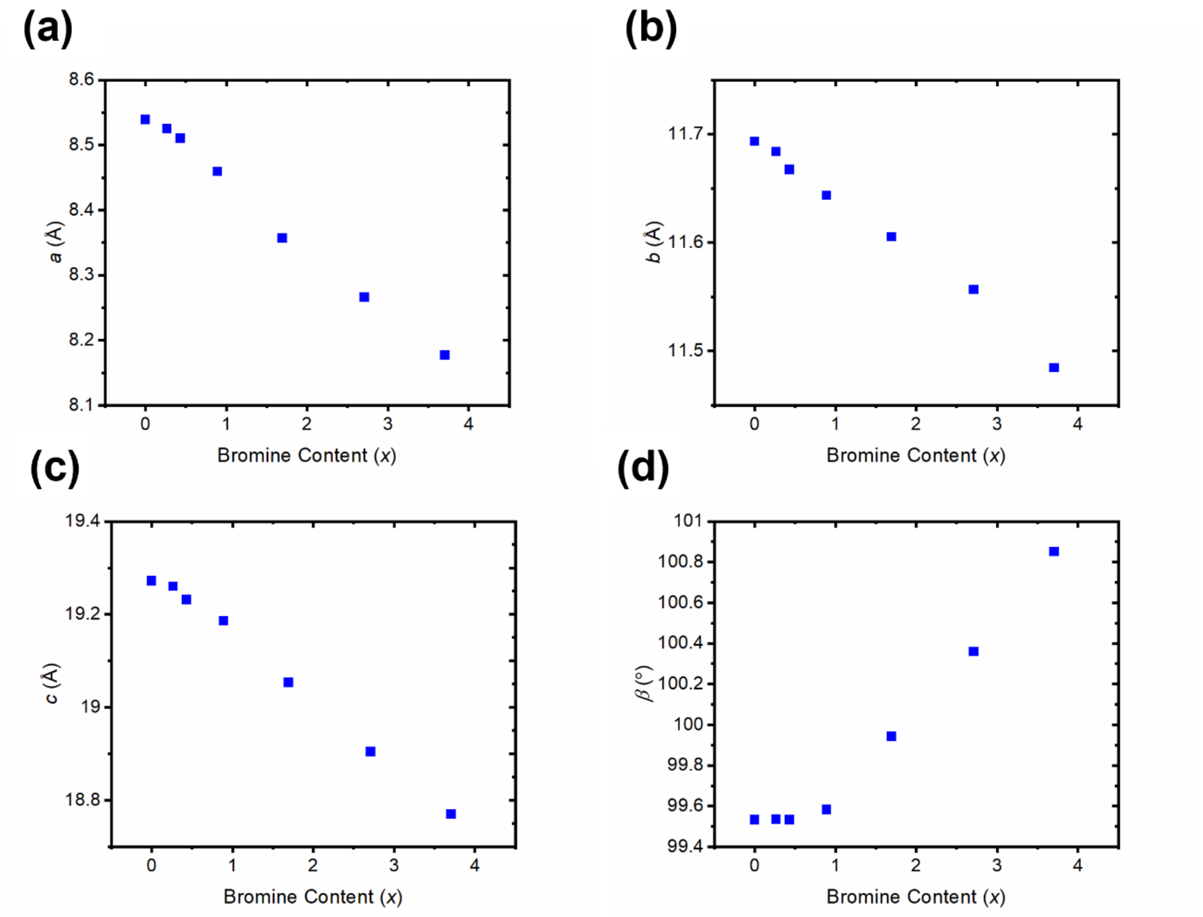

Figure S6: Variation of unit cell parameters with bromine content (*x*) for $MXDBiI_{5-x}Br_x$ samples. The iodine content in the samples is equivalent to 5-*x*, where *x* = Br content. (a) *a* unit cell parameter, (b) *b* unit cell parameter, (c) *c* unit cell parameter and (d) β unit cell parameter. Halide ratios were determined using Energy Dispersive X-ray Spectroscopy using the SEM. When samples were prepared using the synthetic route described in this paper, the *x* = 5 sample could not be isolated with the $MXDBiI_5$ structure, but $MXD_3Bi_2Br_{12}{\cdot}2H_2O$ was obtained instead. Due to the different structure and composition of this material, the data points corresponding to this material have been excluded from these plots.

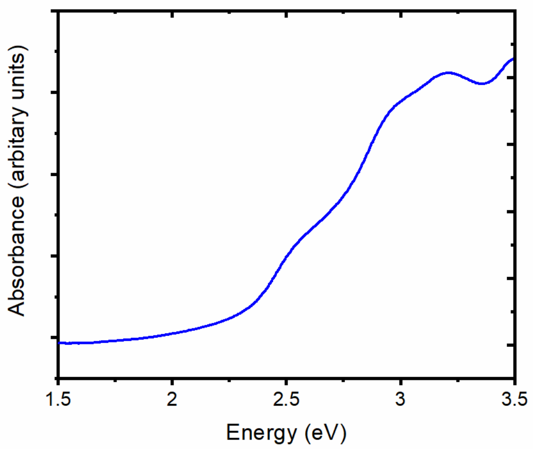

Figure S7: UV-Visible Spectrum of MXD_2PbI_6

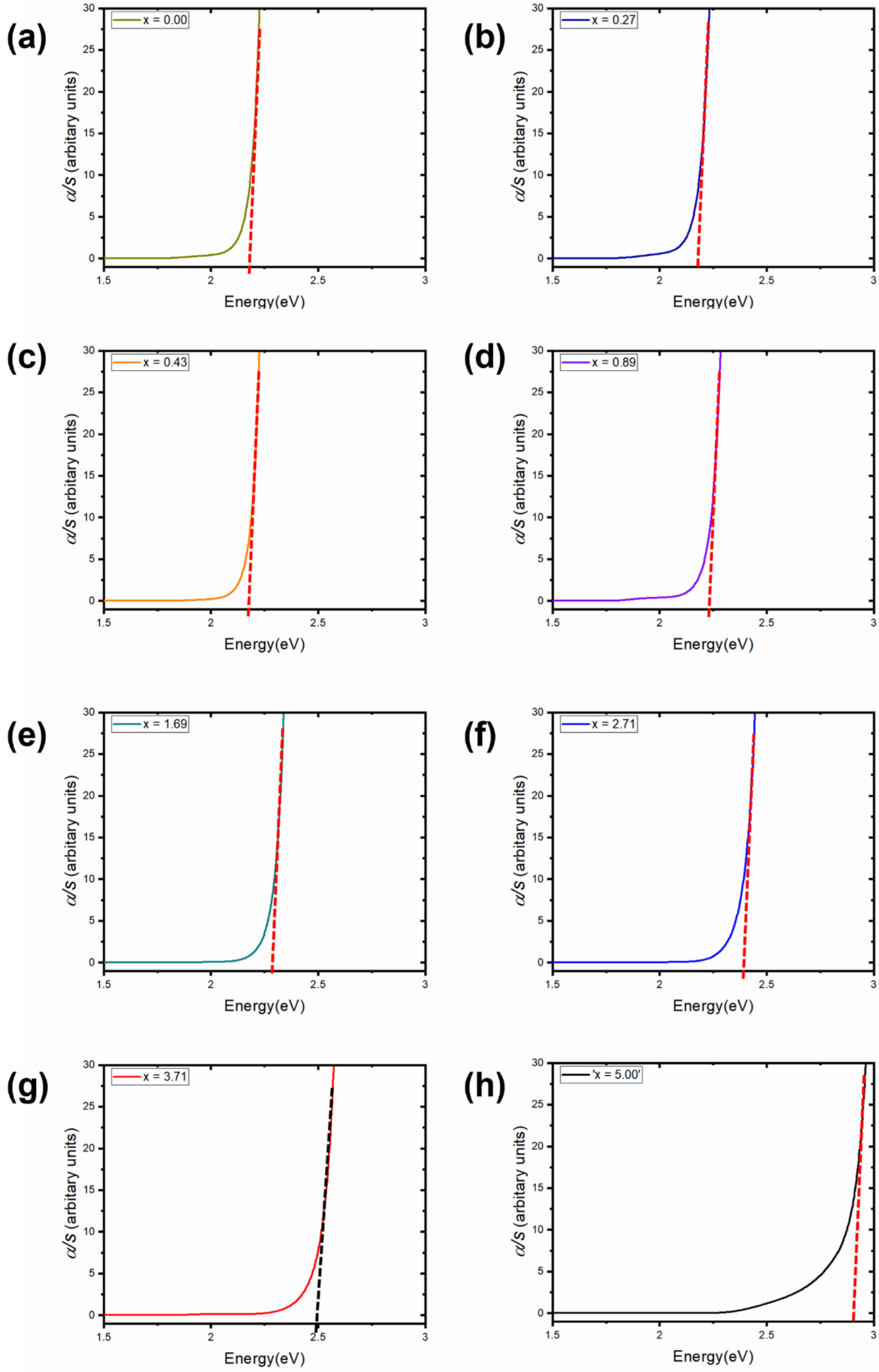

Figure S 8: Fitting of Kubelka-Munk transformation of UV-Visible Diffuse Reflectance Spectra for $MXDBiI_{5-x}Br_x$.
